# High-efficiency upconversion process in cobalt and neodymium doped graphene QDs for biomedical applications

**DOI:** 10.1038/s41598-023-37518-x

**Published:** 2023-06-24

**Authors:** Armin Zarghami, Mahboubeh Dolatyari, Hamit Mirtagioglu, Ali Rostami

**Affiliations:** 1 Photonics and Nanocrystal Research Lab. (PNRL), University of Tabriz, Tabriz, 5166614761 Iran; 2 SP-EPT Lab., ASEPE Company, Industrial Park of Advanced Technologies, Tabriz, Iran; 3 Department of Statistics, Faculty of Science and Literature, University of Bitlis Eren, Bitlis, Turkey

**Keywords:** Biotechnology, Biomaterials, Biomedical materials

## Abstract

Multiphoton absorbing upconversion nanoparticles are emerging as bioimaging materials but are limited by the low quantum yield of their visible fluorescence. This article contains colloids of graphene quantum dots (GQDs), Neodymium, and Cobalt doped Graphene Quantum dots (Co-GQDs and Nd-GQDs) surrounded by carboxylic acids are synthesized which especially are suitable for bio applications; in this way, carboxylic acid groups exchanged by Amoxicillin as an antibiotic with bactericidal activity. The XRD diffraction method, TEM microscope, UV–Vis, and photoluminescence spectroscopies characterize the synthesized materials. The synthesized Quantum dots (QDs) exhibit upconversion properties and their emission is centered at 480 nm, but a red shift was observed with the increase of the excitation wavelength. In the emission spectra of synthesized QDs that can be related to the defect levels introduced by passivation of the QDs in the structure, the results show that with the interaction of the surface QDs with more carboxylic groups, the redshift is not observed. As the results indicate an increase in the intensity of upconversion emission is recorded for Co-GQDs and Nd-GQDs. The absolute quantum efficiency (QY) for Co-GQDs and Nd-GQDs were determined to be 41% and 100% more than GQDs respectively. DFT calculations indicate a strong bond between graphene and cobalt and Neodymium atoms. In doped materials, there are trap levels between the band gap of the GQDs which are responsible for increasing the intensity of the upconversion phenomenon.

## Introduction

In recent years, graphene quantum dots (GQDs) have attracted much attention due to their unique structural and optoelectronic properties and their great potential in various applications in drug delivery^1–5^, sensors^6–12^, bio-imaging^13–18^, magnetic hyperthermia^19–21^, photothermal therapy^22–26^, antibacterial^23–28^, catalyst^29–33^, environmental protection^34,35^, energy conversion, spintronics, photovoltaic devices^36–53^ has made a remarkable accomplishment. One of the important features of these nanoparticles is having high fluorescence properties that can be used for biological imaging and optical sensing. GQDs have attracted much attention as promising biomaterials due to their exceptional advantages of low cytotoxicity, excellent solubility, stable photoluminescence (PL), biocompatibility, and low cost^54–59^.

Conventional imaging probes, such as fluorescent dyes and fluorescent proteins, have attracted much attention. However, they face poor diagnostic sensitivity, instability, and high toxicity and have no therapeutic effect. It was difficult to find a dual-purpose material that could act as an imaging probe and a drug delivery at the same time. As an effective drug delivery, the fluorescent matrix should meet the following criteria: (1) effective drug delivery, (2) good biocompatibility, and (3) good circulation stability in vivo^60–63^. Recently, the rapid development in nanomedicine has opened a new way to find such materials, and nanomaterials have become a very important topic in biomedical applications due to their great potential for the formulation of anticancer drugs. Engineered nanomaterials that have the effect of permeability, biocompatibility, and low toxicity can be used in biomedical applications, including biomedical diagnosis and disease treatment^64,65^.

Up conversions, nanoparticles (UCNPs) have anti-Stokes luminescence that can convert near-infrared (NIR) light into visible light. UCNPs have high chemical stability and can be used as fluorescence probes in a variety of complex organisms and facilitate the bioassay process. In addition, UCNPs can lead to surface conjugation with specific targeting ligands (such as peptides, antibodies, and small molecule drugs) and can be used as probes to identify and target specific cells with high sensitivity and selectivity^66,67^. Lanthanide-doped materials are among the most well-known UCNPs in which trivalent lanthanide ions are dispersed as guests in a suitable host network with dimensions less than 100 nm. Lanthanide impurities are photoactive centers that emit light after excitation with low-energy photons. Through a suitable selection of lanthanide dopants, UCNPs can exhibit selective wavelength (color) conversion such as NIR to shorter NIR, visible (blue, green, red), or UV^68^.

UCNPs are an improved alternative to traditional optical imaging materials due to several other advantages, such as weak photobleaching, low background fluorescence, deep tissue penetration, and minimal photodamage^69–73^. In addition, as a new generation of imaging agents, UCNPs are widely used in PDT, MRI, X-ray CT imaging, photoacoustic imaging (PA), NIR thermal imaging, phototherapy (PTT), chemotherapy, and radiotherapy. Increasingly studies have focused on their application in PDT because they can act as a photosensitizer in photodynamic therapy^74–79^. Also, they can be used as drug carriers for bioimaging therapeutic drugs or genes (such as doxorubicin, siRNA, DNA, and microRNA) or light-sensitive materials. More importantly, UCNP in antibacterial photodynamic therapy (aPDT) has played an essential role in the treatment of bacterial infectious diseases, such as periodontitis and Staphylococcus aureus infections, which are incurable and difficult to eradicate^80^. The development of nanomaterials that exhibit both good upconversion luminescence (UCL) performance and good performance is undoubtedly a challenge for materials scientists, physicists, and chemists.

Surface modification of nanoparticles is usually necessary to create a suitable surface composition for biomedical applications^81^. For example, surface modification of UCNPs is important for loading some hydrophobic drugs, and surface charge for the adsorption of small molecules^82^. However, different needs make different changes, and careful design and optimization of these aspects are necessary before using UCNPs for bioanalytical applications^83^.

Bioconjugation of UCNP materials with biomolecules is very important in therapeutic and diagnostic applications. Recent research has shown that surface-modified ligands on UCNPs can be transformed into a new functional group to influence the subsequent bioconjugation step. Ligands commonly used for coupling are maleimides^84^, thiols^85^, carboxylic acids^86^, aldehydes, and amine groups.

Compared with traditional medicine, UCNP-based drug delivery has obvious advantages such as small particle size, which facilitates the endocytosis of cells to obtain a good therapeutic effect^60^. The large surface area of UCNPs can prolong the shelf life of topical drugs and enhance the use of drug targeting in tissues^87,88^. Even better, UCNP-based composites used as drug delivery systems allow real-time tracking and evaluation of drug release efficiency^89,90^.

Graphene-based nanomaterials have been designed to help deliver or target drugs more effectively. They are being investigated for therapeutic applications, especially for cancer treatment, as well as for the development of new diagnostics and nanosensors, and are expected to contribute to molecular imaging for diagnosis and treatment, especially in the development of therapeutic strategies in oncology^91–93^.

In this article, colloidal graphene nanoparticles doped with cobalt and neodymium cations were designed and synthesized with the aim of biological imaging. The surface of nanoparticles is decorated by carboxylic groups that can be bioconjugated with biomolecules. Here our goal is to increase the quantum yield of the upconversion property of the graphene quantum dots by doping cobalt and neodymium ions in their structure. The results show a 41% increase for cobalt and a 100% increase for neodymium-doped graphene in the intensity of the upconversion phenomenon. The mentioned structures were simulated using DFT calculations and their electronic properties were obtained.

## Materials and method

### Chemicals

All chemicals purchased from Merck Company and used as received.

### Synthesis of cobalt-doped GQDs

The GQDs and Co-doped GQDs were prepared by the thermal decomposition of citric acid (CA). In a typical procedure of GQDs preparation, 2 g CA and, 0.1 g of the cobalt acetate was put into a 50 mL beaker and heated to 200 °C using a heating mantle. About 5 min later, the CA was liquated. Subsequently, the color of the liquid was changed from colorless to pale yellow, and then orange in 30 min, implying the formation of GQDs. The obtained orange liquid for preparing GQDs was added drop by drop into 100 mL of 10 mg mL^−1^ NaOH solution, under vigorous stirring. After neutralizing to pH 7.0 with NaOH, the aqueous solution of GQDs was obtained^94^. For synthesis, Nd-doped Graphene cobalt acetate was substituted by Neodymium acetate. The obtained synthesized materials were centrifuged and washed with water for 1 h at 12,000 rpm and redispersed in deionized water.

### Exchanging amoxicillin (6-[D(−)β-amino-p-hydroxyphenyl-acetamido]) with surface carboxylic acids

0.5 g of Amoxicillin was solved in 10 CC of deionized water and dropwise added on 20 CC of the synthesized colloids and stirred for 2 h. The obtained exchanged materials were centrifuged and washed with water for 1 h at 12,000 rpm and redispersed in deionized water.

### Characterization

The size and shape of synthesized nanoparticles were measured at ×200–×300 magnifications using TEM microscopy imaged by a Zeiss-EM10C TEM microscope at 80 kV accelerating voltage and ~ 2 nA beam current. The EDX analysis was employed to identify the elemental composition of the nanoparticles. UV–Vis absorption spectra were recorded employing a PG Instruments Ltd T70 UV/Vis spectrophotometer. PL spectra were taken by a Perkin Elmer LS-45 spectrophotometer equipped with a 500 W Xenon lamp. For lower powers, we used an optical filter in front of the instrument’s source. ICP analysis was recorded by ICP-OES SPECTRO ARCOS. Powder X-ray diffraction patterns were recorded by XRD Bruker D8 advance**.**

### DFT simulations

The Gaussian 03 package and materials studio CASTEP model was applied for DFT calculation. The electronic structures were established based on Graphene nanoclusters containing 54 carbon atoms passivized by carboxylic groups and bonded with Cobalt acetate surrounded by H_2_O molecules. B3LYP/6-311G (d,p) basis sets and LDA approximation were used in the geometry optimizations. In the CASTEP, calculation, the number of plane waves included in the basis was determined from cut-off energy (*E*c) of 500.0 eV. The summation over the Brillouin zone was carried out with *k-*point sampling using a Monkhorst–Pack grid. Geometry optimization under applied hydrostatic pressure with parameters of 2 × 2 × 5 was used to determine the modulus of a material (B) and its pressure derivative, Bʹ = dB/dP^95^.

## Results and discussion

**Figure 1:**
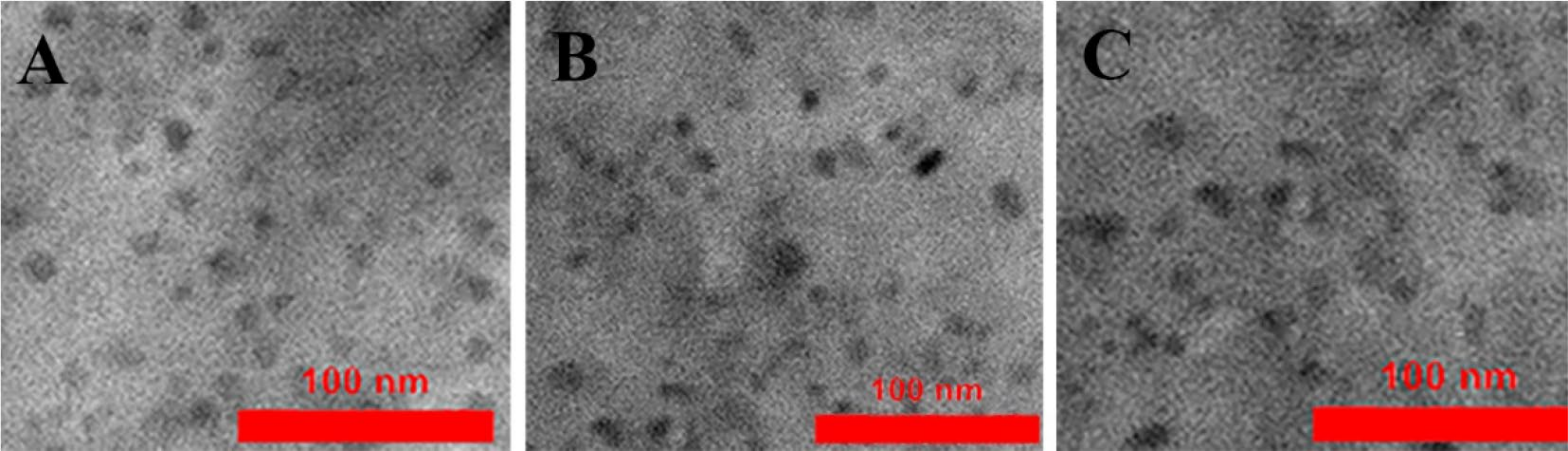
TEM image of the synthesized (**A**) GQDs (**B**) Co-GQDs (**C**) Nd-GQDs.

Figure 1 shows the TEM image of the synthesized GQDs and their doping with cobalt and Neodymium ions with a diameter of 10 nm. As the figure shows the shape and size of quantum dots are nearly the same for all materials.

**Figure 2:**
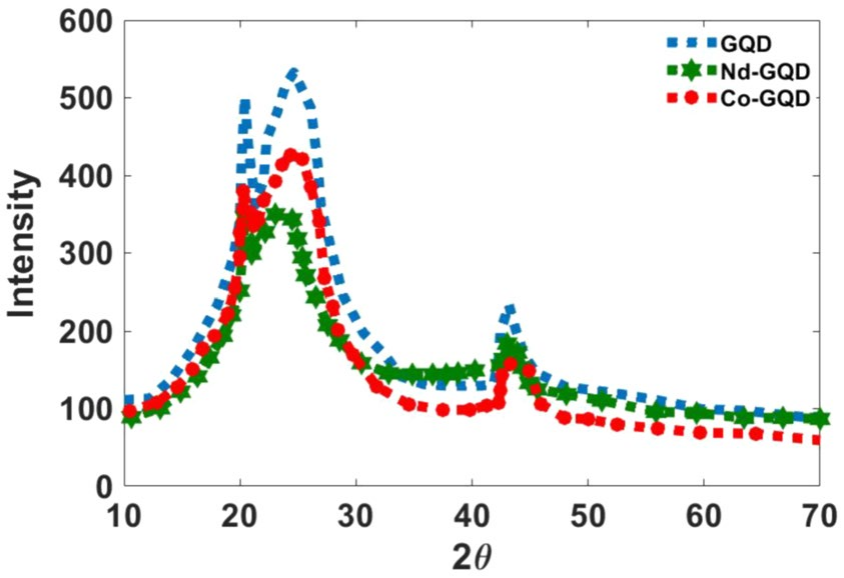
The XRD diffraction pattern for synthesized CO-Graphene and Nd-Graphene quantum dots.

X-ray diffraction shows that no peak related to cobalt and Nd ions can be seen and only there are small shifts in the appeared peaks in the related patterns, which indicates the doping of ions in the structure of the graphene quantum dot. The peaks appeared at 2θ = 24° along the (002) orientation and 2θ = 43° according to (102) orientation indicating synthesizing GQDs (see Fig. 2). As the figure shows crystallinity in doped graphenes is less than in undoped Graphene.

ICP measurements indicate 10 and 12 ppm Co and Nd ions in the colloidal sample respectively.

**Figure 3:**
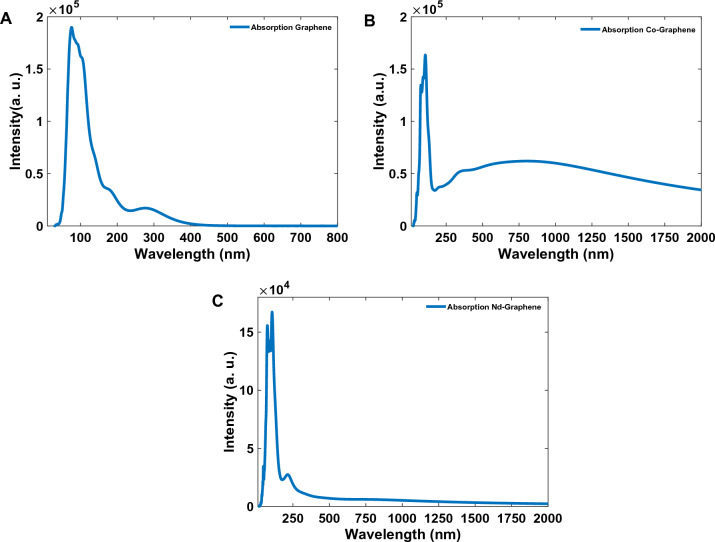
Calculated absorption spectra of (**A**) GQDs, (**B**) CO-GQDs, and (**C**) Nd-GQDs.

Figure 3 shows calculated absorption spectra for GQDs, Co-GQDs, and Nd-GQDs. As Fig. 3A shows there are peaks centered at 74.3, 179.6, and 281.2 nm for GQDs, and in wavelengths, upper than 400 nm the absorption intensity is zero. For Co-doped graphene QDs, the observed peaks are at 75.56, 106.6, and 315 nm, and 74.02, 107.3, and 220.6 nm for Nd-doped QDs. Also in doped materials, there is absorption at wavelengths upper than 400 nm. This means there are created trap electronic levels between the band gap of the graphene after doping cobalt and neodymium ions in the structure of graphene.

**Figure 4:**
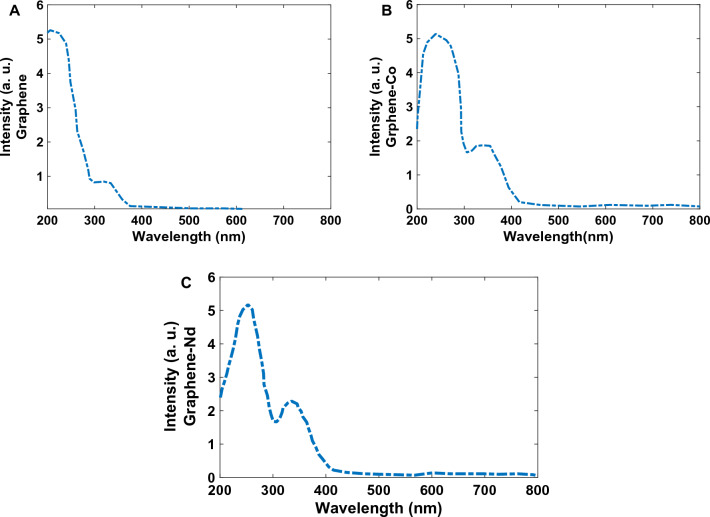
Absorption spectra of (**A**) GQDs, (**B**) Co-GQDs, and (**C**) Nd-GQDs.

The absorption spectra of synthesized GQDs, Co-GQDs, and Nd-GQDs show two absorption bands. The first peak for Graphene is at 211.11 nm and the second one is at 324.4 nm (Fig. 4A). The first peak for Co-doped graphene is at 244 nm and the second peak is at 338.9 nm (Fig. 4B). The first peak for Nd is at 250 nm and the second is at 331.1 nm (Fig. 4C). The peaks are relatively broad and we can’t recognize other peaks related to electronic levels of Co and Nd ions. Shifts in both peaks can be related to differences in the radius of doped ions and the effect on the electronic structure of Graphene. A comparison of the GQDs without Cobalt atoms indicates that the intensity of the peak at 350 nm is high in cobalt-doped graphene. Also, the absorption intensity in wavelengths upper than 400 nm is not zero for doped synthesized materials.

To further investigate the optical properties of the synthesized GQDs, a detailed PL study was performed using different excitation wavelengths. In general, the PL spectrum of graphene nanoparticles depends on the excitation wavelength. In other words, the PL peaks shift to longer wavelengths of maximum intensity because the excitation wavelength is a bathochromic shift^96^. Shen et al.^97^ have reported that the linear relationship between E_m_ and E_x_, and the function of the fit line is E_m_ = 1.00E_x_ + δE (R^2^ = 0.9983) with δE = 1.1 eV. All these changes come from the surface passivation of nanoparticles (in our case citrate ligands) that strongly influence the optical properties of the GQDs and trap states created by surface defects of the structure.

**Figure 5:**
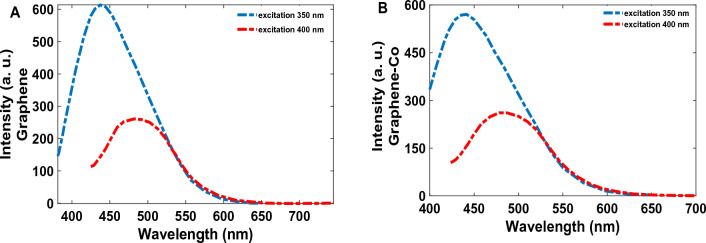
PL spectra of (**A**) GQDs and (**B**) Co-GQDs, excited at 350, and 400 nm.

The photoluminescence emission spectrum of the synthesized Co-GQDs and GQDs at 350 and 400 nm excitation wavelengths are presented in Fig. 5. The observed emission bands for both synthesized GQDs and Co-GQDs are at 450 and 480 nm (excitation wavelengths are 350 and 400 nm). Also, a decreasing trend in emission intensity values was observed with increasing excitation wavelength.

As the figure shows, the intensity of photoluminescence in Graphene is relatively higher than Co-GQDs because of inserted trap levels by doping Co atoms (it will be described more in the mechanism section).

**Figure 6:**
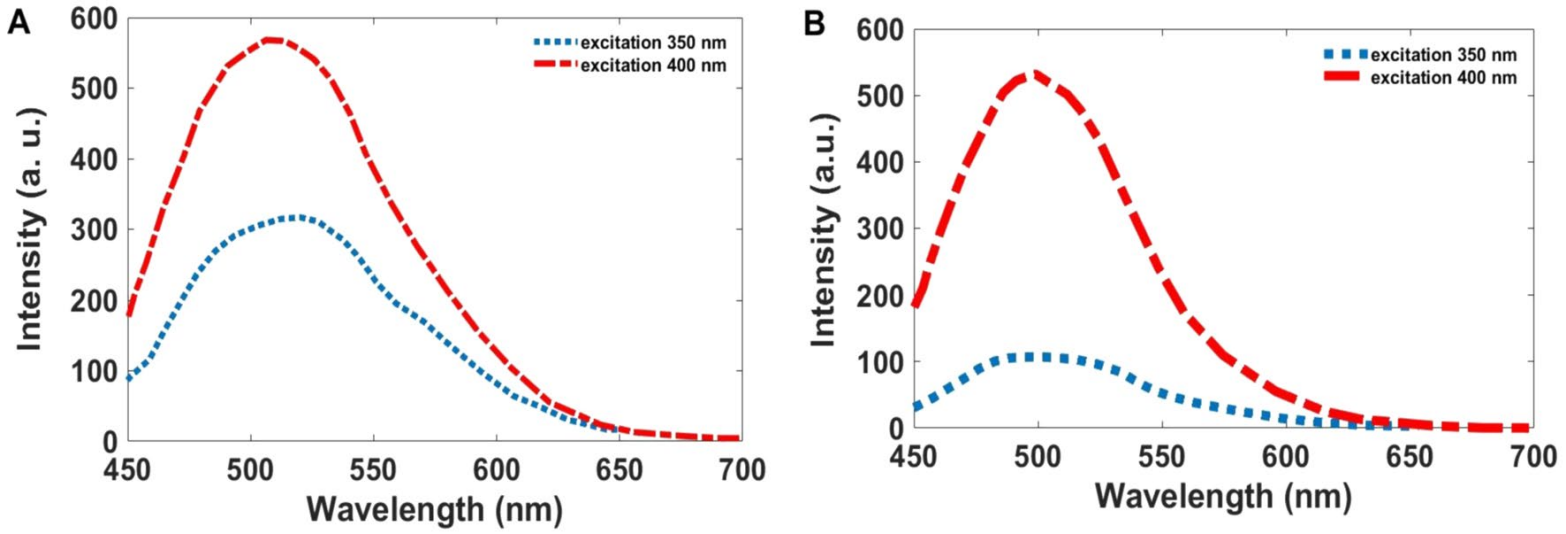
PL spectra of (**A**) GQDs and (**B**) Co-GQDs (surface-modified particles), excited at 350, and 400 nm.

To prove and correct this phenomenon, the surface of nanoparticles was modified with excess citric acid (how to do the work is explained in the materials and methods section). As Fig. 6 shows, nanoparticles modified with citric acid do not show any frequency shift at 350 and 400 nm excitation, and the emission peak is observed at 500–550 nm.

**Figure 7:**
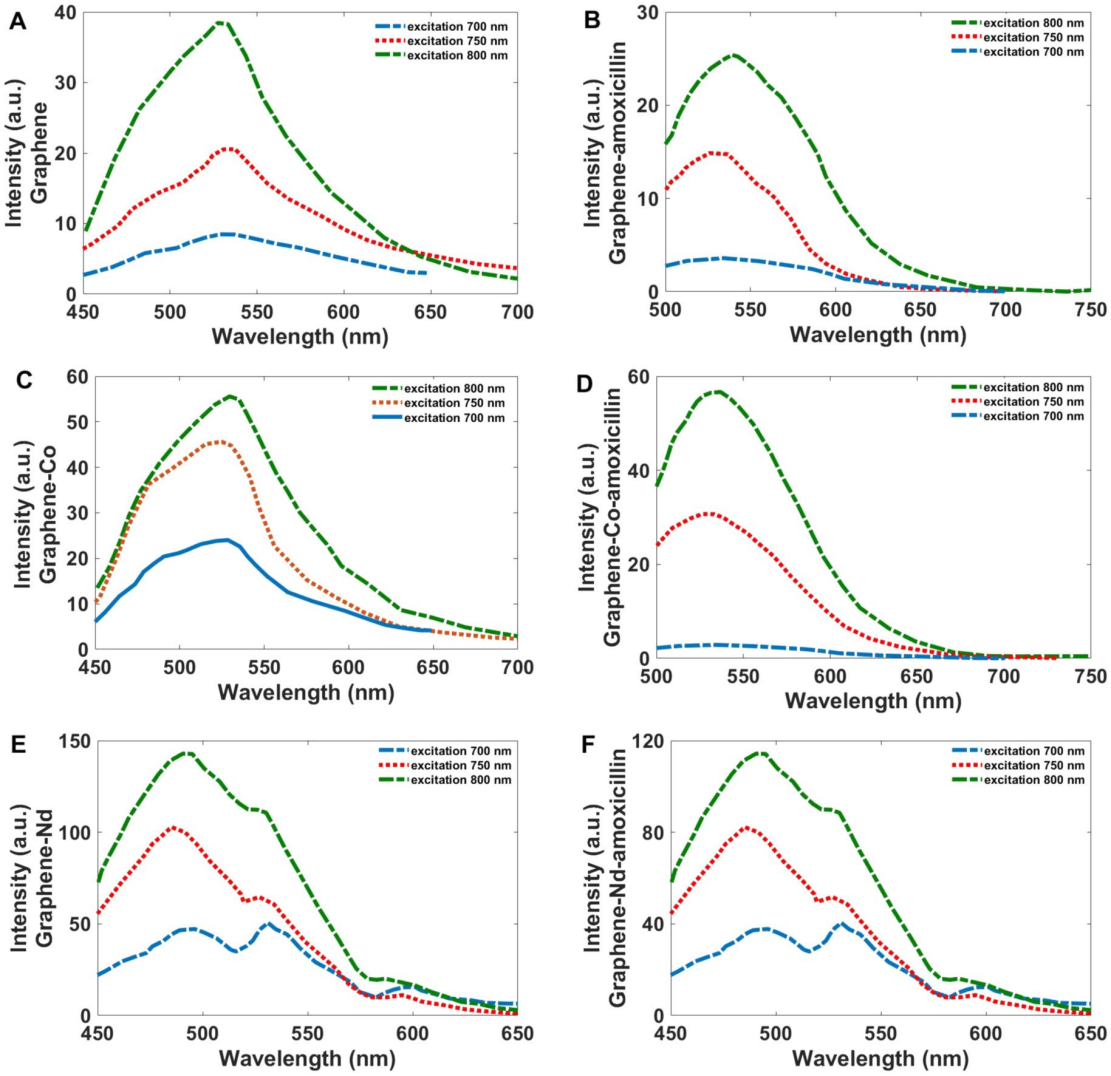
PL spectra of (**A**) GQDs, (**B**) Co-GQDs, and (**C**) Nd-GQD, excited at 700, 750, and 800 nm.

In addition to the PL features, significant upconversion is observed for the synthesized Co-GQDs and Nd-GQDs. Figure 7 shows the PL spectra of the GQDs, Co-GQDs, and Nd-GQDs synthesized nanoparticles excited by long-wavelength light at 650, 700, 750, and 800 nm. The PL spectra show a strong peak centered at 525 nm for both GQDs and Co-GQDs with an excitation wavelength of 800 nm. For Nd-GQDs, there is a strong peak at 500 nm and a shoulder centered at 530 and 600 nm that can be attributed to the electronic transition from ^4^G_7/2_ + ^2^K_13/2_ and ^2^G_7/2_ + ^4^G_5/2_ to ^4^I_9/2_ respectively^98^. As the figure shows an increase in the intensity of PL spectra is recorded for Co-GQDs and the absolute quantum efficiency (QY) for Co-GQDs, and Nd-GQDs were determined to be respectively 41% and 100% more than Graphene QDs. As Fig. 7B,D,F show, binding a biomolecule (Amoxicillin) on the surface of synthesized nanoparticles decrease the intensity of upconversion emission; however, this increase is negligible and the synthesized materials can act as drug delivery compounds.

**Figure 8:**
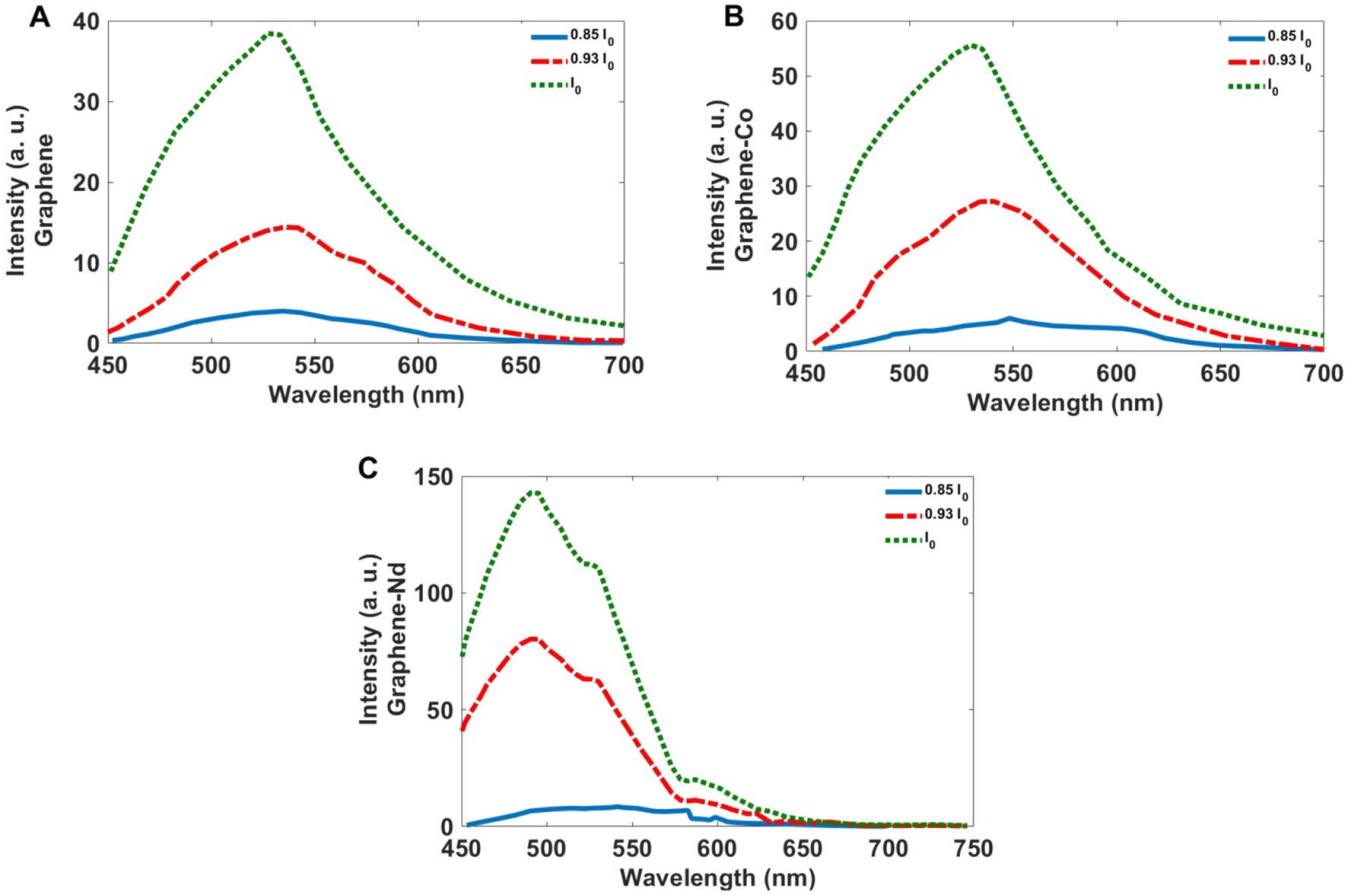
PL spectra of (**A**) GQDs, (**B**) Co-GQDs, and (**C**) Nd-GQD, excited 800 nm for a source with the power of I_0_, I_0_/2, I_0_/3.

The upconversion effect of nanoparticles was examined by different power sources (Fig. 8). The obtained PL spectra indicate with the decrease in power source the same bands with lower intensity are observable.

**Figure 9:**
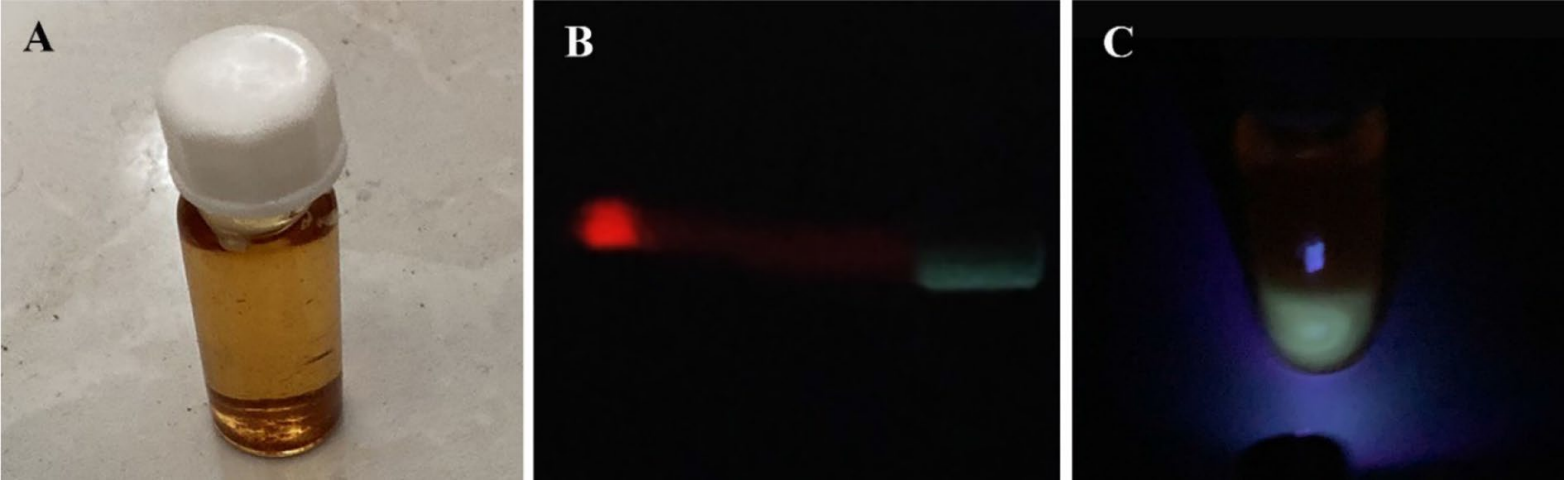
Up and down conversion effect for synthesized Nd-GQD (**A**) synthesized Nd-GQDs (**B**) Nd-GQD under excitation of a laser with wavelength 750 nm, 1w (**C**) Nd-GQD under LED with wavelength 350 nm, 1mw.

Figure 9 shows the up-and-down conversion effect of synthesized Nd-QDs under the excitation of a laser with a wavelength of 750 nm and an LED with a wavelength of 400 nm.

### Mechanism of upconversion transition

**Scheme 1:**
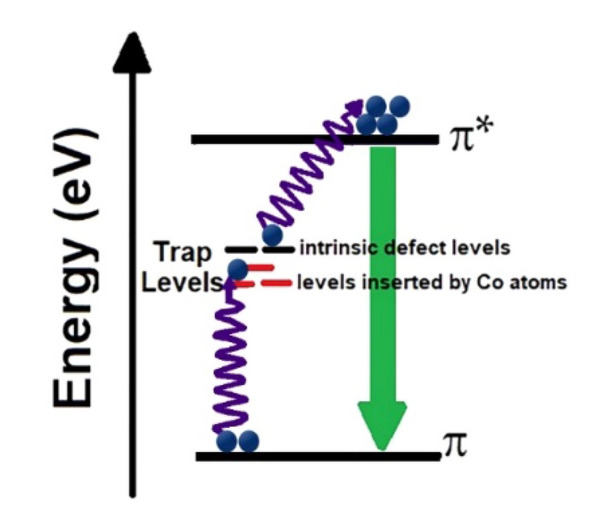
A simple energy diagram of optical transition states for illustrating the upconversion phenomenon in the synthesized Co-GQDs and Nd-GQDs.

A simplified energy diagram of optical transition states for cobalt and Neodymium doped graphene structure to illustrate the upconversion mechanism is shown in Scheme 1. As shown in the figure, the presence of levels between HOMO and LUMO in graphene is introduced by doped atoms in the electronic structure and they are related to the unfilled *d* orbitals of Cobalt ions and *f* orbitals related to Neodymium, allowing the absorption of photons with lower energy. It means that absorbing multi-photons with lower energy can excite electrons in multi-steps, which increases the intensity of upconversion emission in cobalt-doped graphene. These trap levels can also reduce the intensity of photoluminescence emission (excitation wavelengths: 350 and 400 nm) in doped quantum dots and increase non-radiative transitions in this structure.

### HOMO/LUMO calculations

**Figure 10:**
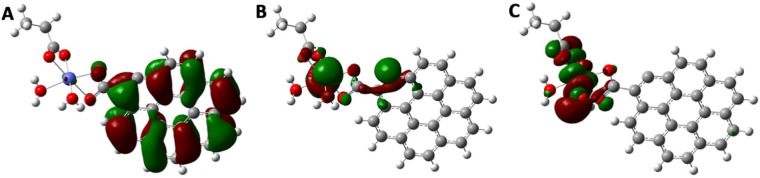
DFT Calculated (**A**) HOMO (**B**) SOMO (**C**) LUMO molecular orbitals of the synthesized Co-GQDs.

Figure 10 illustrates HOMO/LUMO orbitals of the Cobalt doped graphene sheet (G) which was computed at B3LYP/6-31G(*d*,*p*) theoretical level. HOMO/SOMO/LUMO orbitals of the Cobalt doped graphene sheet, show highly symmetric configurations indicating the homogeneity of electronic distribution through these orbitals either in the ground states on carbon atoms of the graphene sheet. However, they present asymmetric distribution for SOMO and LUMO, and orbitals are nearly concentrated around the Graphene's added Cobalt atoms and carboxylic acid groups.

**Figure 11:**
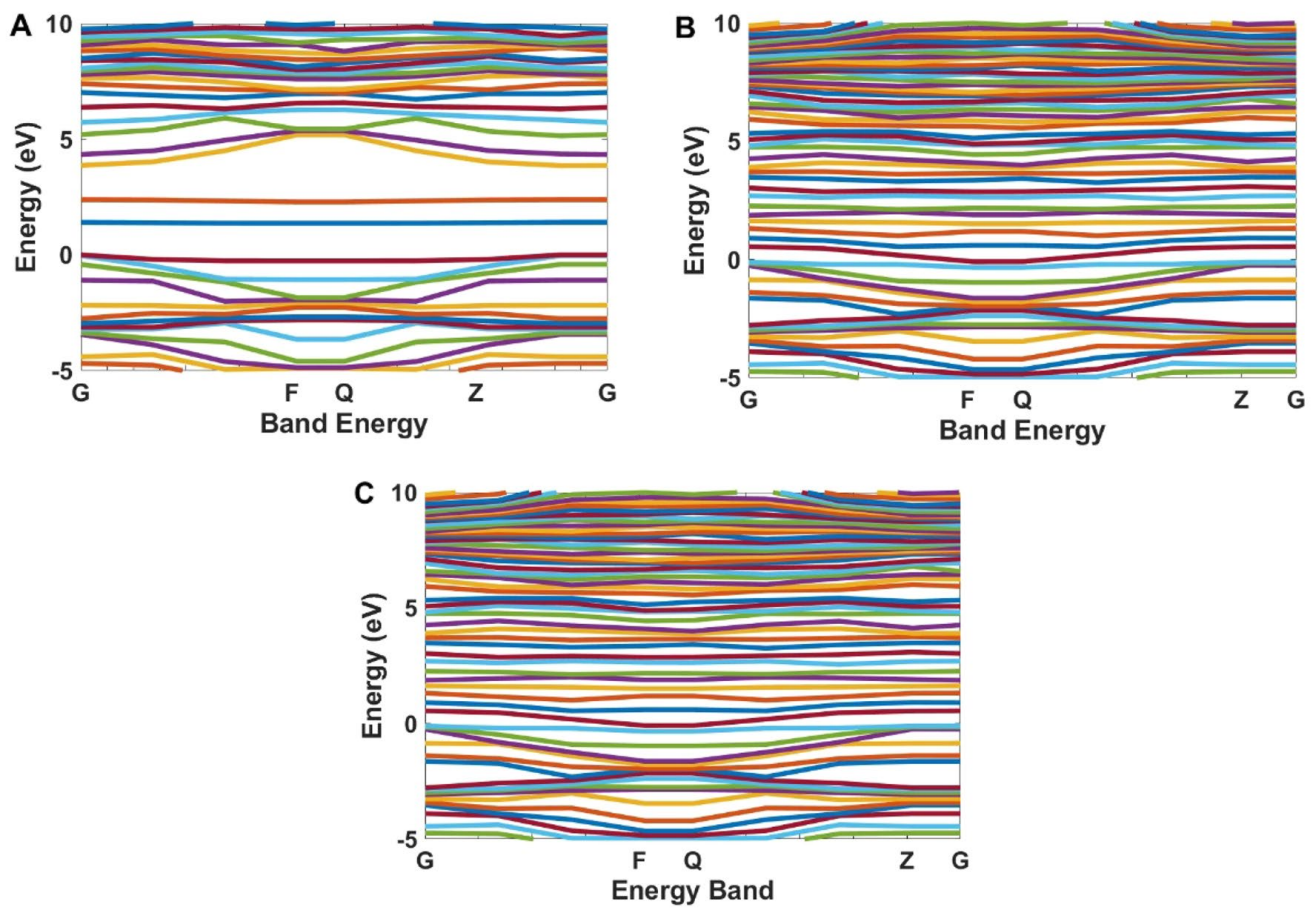
Band structure calculation for (**A**) GQDs, (**B**) Co-GQDs, and (**C**) Nd-GQD.

Calculated band structures for GQD, Co-GQD, and Nd-GQD are presented in Fig. 11. This figure indicates that the band gap energy for GQD is 2.41 eV (514 nm), which agrees well with experimental results. After doping Cobalt and Neodymium some electronic bands are created between the band gap. These levels are denser in Neodymium doped Graphene.

**Figure 12:**
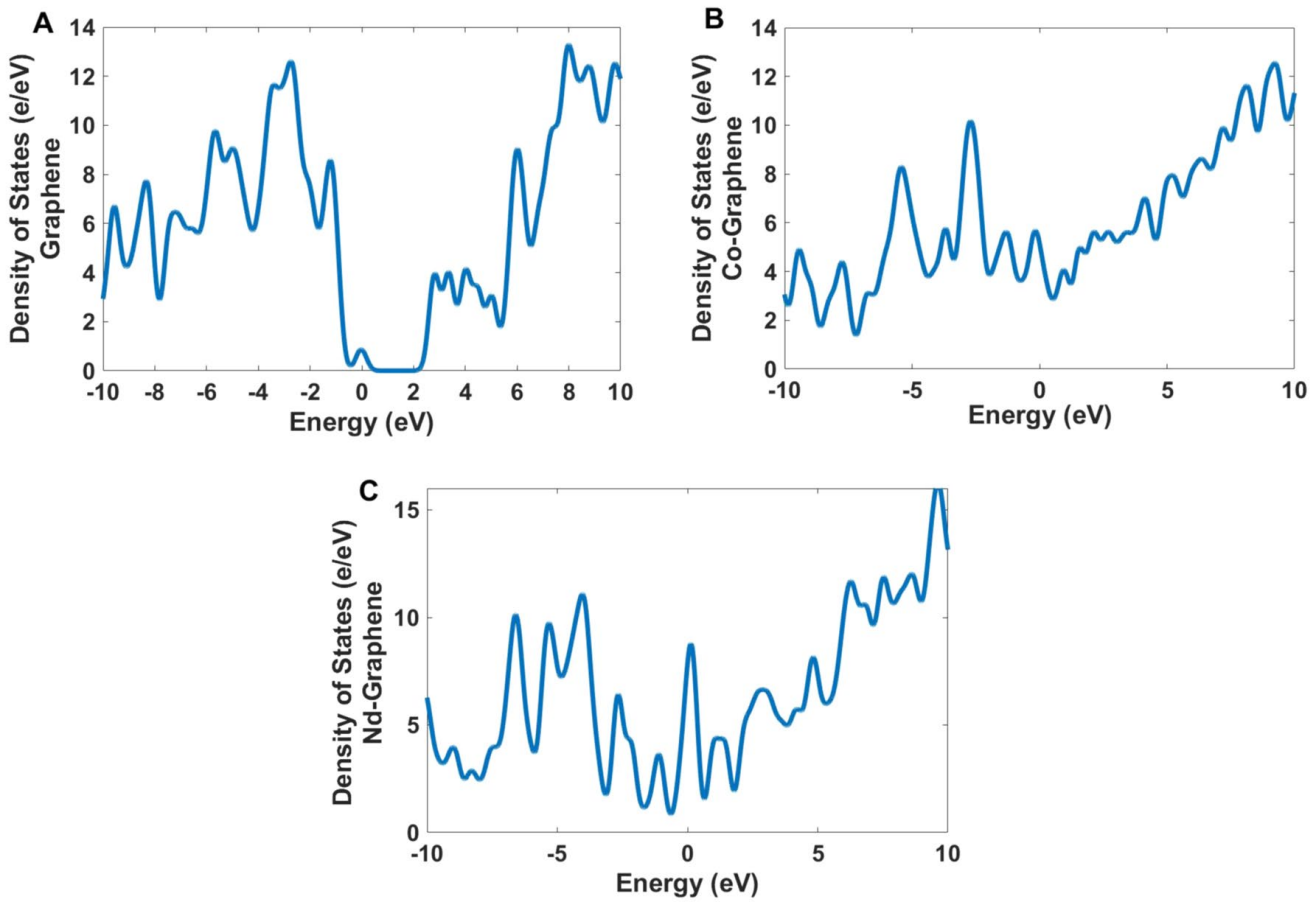
The density of States for (**A**) GQDs, (**B**) Co-GQDs, and (**C**) Nd-GQD.

**Figure 13:**
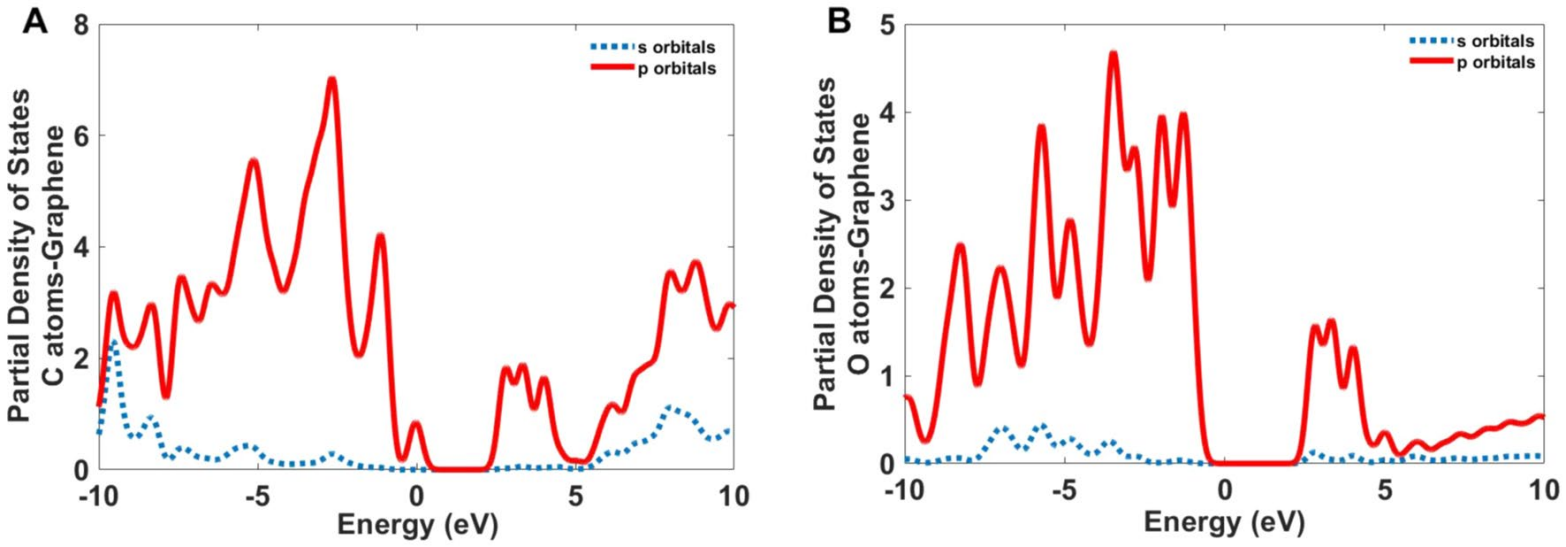
The partial density of states for the C and O atoms of the GQDs.

**Figure 14:**
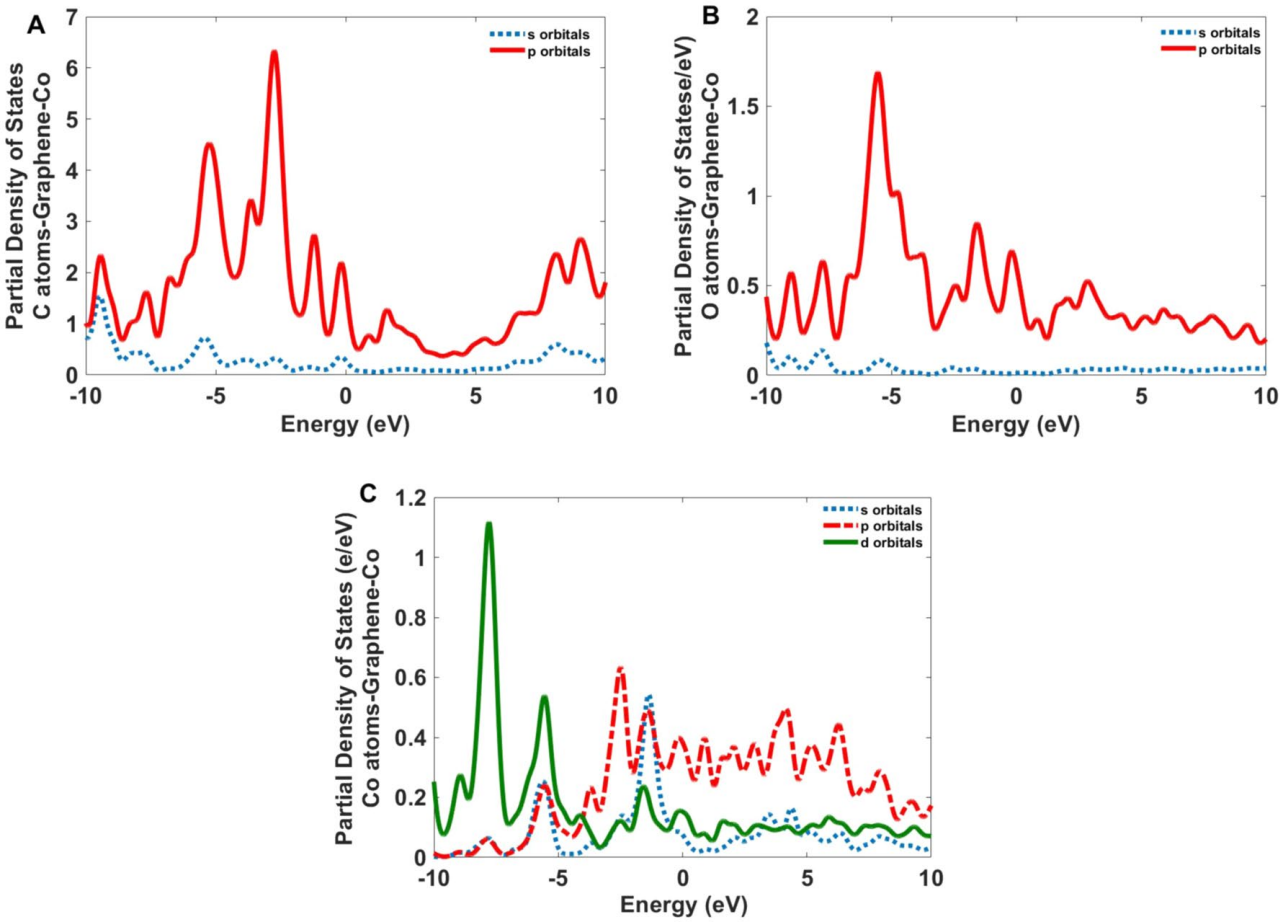
The partial density of states for the C, O, and Co atoms of the Co-GQDs.

**Figure 15:**
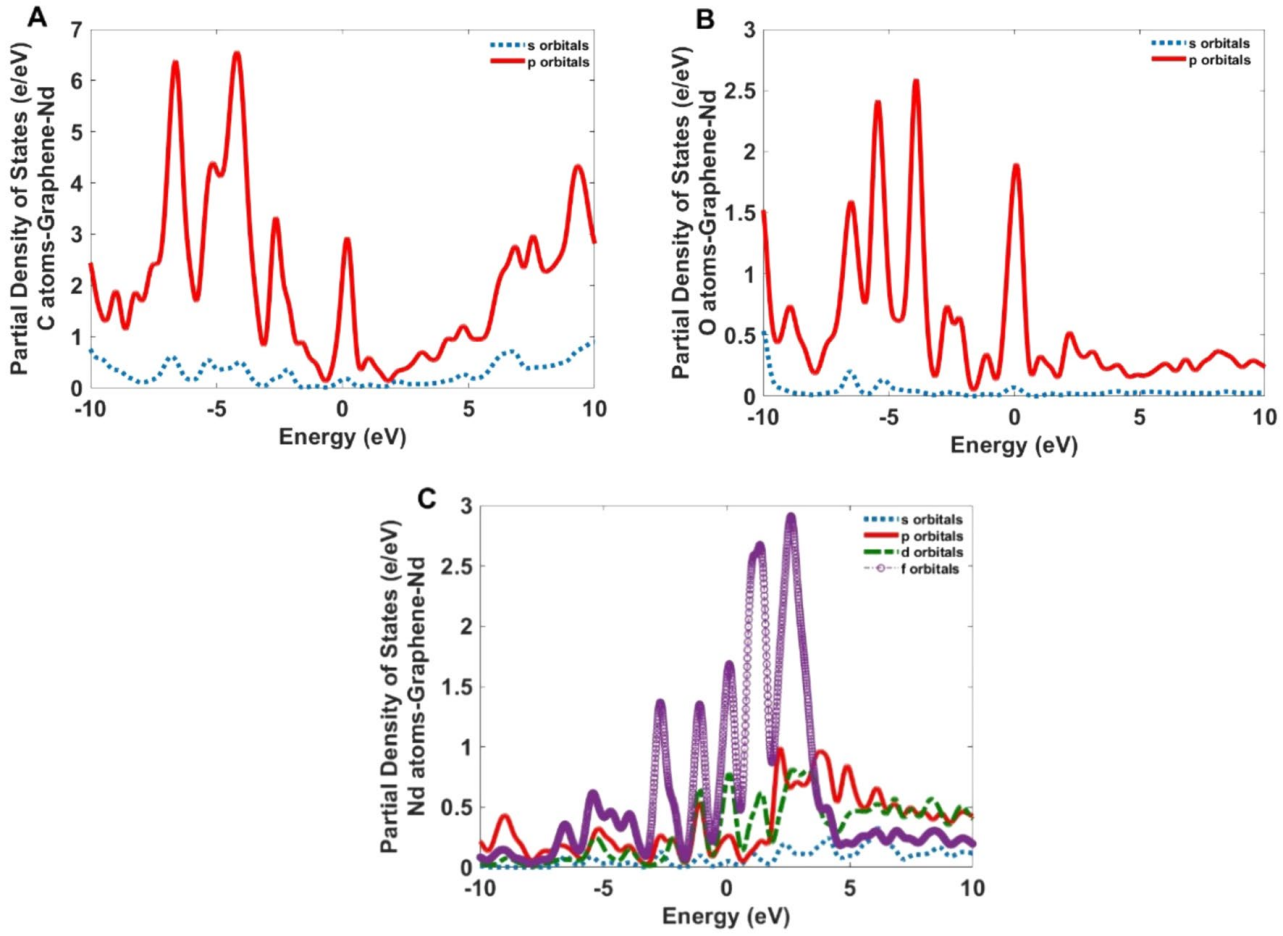
The partial density of states for the C, O, and Nd atoms of the Nd-GQDs.

The density of states (DOS) in Fig. 12 shows that there is a big difference between intrinsic graphene and the Co and Nd doped graphene between 0 and 2 eV. Figures 13, 14 and 15 illustrate the contribution of orbitals in electronic transitions for GQD, Co-GQD, and Nd-GQD.

As described above, there are carboxylic groups around Graphene QDs and we calculated the partial density of states for C and O atoms. As Fig. 13 shows the *p* orbitals of C and O atoms are attributed to the electronic transitions of GQDs.

Figure 14 indicates the *p* orbitals of the C and O atoms also, the *p* and *d* orbitals of the Co atoms have the most contribution to electronic transitions of Co-GQD. For Nd-GQD (Fig. 15), the *p* orbitals of C and O atoms also *f* orbitals of Nd atoms have a high contribution to the electronic transitions.

## Conclusion

In this article, the colloids of Graphene, Graphene-cobalt, and Graphene-Neodymium quantum dots decorated by carboxylic groups were synthesized for bio-imaging purposes and their physical properties were characterized. X-ray diffraction results show that cobalt and Neodymium were doped in the graphene structure and DFT calculations confirm that a strong bond was formed between doped atoms and graphene passivized by citrate. Also, the PL spectra indicate the existence of trap levels between HOMO and LUMO levels of Graphene QDs created by doped atoms in the structure. In this condition, multi-photons with lower energies can excite the electrons and the upconversion transitions can be done with higher intensity. As the results show with doping cobalt and neodymium atoms in the structure of graphene, upconversion intensity can be increased to 41% and 100% more than un-doped graphene. By exchanging the carboxylic groups on the surface of synthesized nanoparticles with Amoxicillin as an antibiotic molecule the upconversion property remains and therefore they can use as drug delivery materials in bio-applications.

## Data Availability

The datasets used and/or analyzed during the current study are available from the corresponding author upon reasonable request.
